# Association of Maternal Body Mass Index With Risk of Infant Mortality: A Dose-Response Meta-Analysis

**DOI:** 10.3389/fped.2021.650413

**Published:** 2021-03-12

**Authors:** Nana Huo, Kun Zhang, Li Wang, Lina Wang, Wenhui Lv, Wenke Cheng, GuangZhu Jia

**Affiliations:** ^1^Department of Obstetrics, Maternity and Child Health Care of Zaozhuang, Zaozhuang, China; ^2^Department of Obstetrics and Gynecology, Maternity and Child Health Care of Zaozhuang, Zaozhuang, China; ^3^Department of Cardiology, Heart Center Leipzig at University Leipzig, Leipzig, Germany

**Keywords:** infant, maternal, body mass index, mortality, meta-analysis

## Abstract

**Objective:** This study presumed that a high or low body mass index (BMI) might increase the risk of infant mortality. Therefore, a meta-analysis was performed to systematically assess the association between maternal BMI and the risk of infant mortality.

**Methods:** The electronic databases, including Pubmed, Embase database, and Cochrane Library, were systemically searched by two investigators from inception to November 26th, 2020, with no language restriction. In parallel, a dose-response was assessed.

**Results:** Finally, 22 cohort studies involving 13,532,293 participants were included into this paper, which showed that compared with normal BMI, maternal overweight significantly increased the risks of infant mortality [risk ratio (RR), 1.16; 95% confidence interval (CI), 1.13–1.19], neonatal mortality (RR, 1.23; 95% CI, 1.08–1.39), early neonatal mortality (RR, 1.55; 95% CI, 1.26–1.92) and post-neonatal mortality (RR, 1.18; 95% CI, 1.07–1.29). Similarly, maternal obesity significantly increased the risk of infant mortality (RR, 1.55; 95% CI, 1.41–1.70), neonatal mortality (RR, 1.55; 95% CI, 1.28–1.67), early neonatal mortality (RR, 1.37; 95% CI, 1.13–1.67), and post-neonatal mortality (RR, 1.30; 95% CI, 1.03–1.65), whereas maternal underweight potentially decreased the risk of infant mortality (RR, 0.93; 95% CI, 0.88–0.98). In the dose-response analysis, the risk of infant mortality significantly increased when the maternal BMI was >25 kg/m^2^.

**Conclusions:** Maternal overweight or obesity significantly increases the risks of infant mortality, neonatal mortality, early neonatal mortality, and post-neonatal mortality compared with normal BMI in a dose-dependent manner. Besides, maternal underweight will not increase the risk of infant mortality, neonatal mortality, early neonatal mortality, or postneonatal mortality; instead, it tends to decrease the risk of infant mortality. Early weight management may provide potential benefits to infants, and more large-scale prospective studies are needed to verify this finding in the future.

## Introduction

Obesity may lead to poor maternal and neonatal health outcomes (1). Obesity is more prevalent in women of reproductive age than in the general population. It has been reported that the prevalence of obesity among women aged 20–39 years increases from 31 to 36% from 2007–2008 to 2015–2016 (2), and such changing demographics represent a new epidemiological trend of particular concern to pregnant women. As recommended by the World Health Organization (WHO), body mass index (BMI) is classified as underweight (BMI<18.5 kg/m^2^), normal weight (BMI 18.5–24.9 kg/m^2^), overweight (BMI 25–29.9 kg/m^2^) or obese (BMI ≥ 30 kg/m^2^) (3). Obese and pregnant women are more likely to develop intrauterine and fetal complications as well as maternal health problems; meanwhile, obesity during pregnancy may be significantly negatively associated with fetal and maternal health outcomes, including hyperemesis, pre-eclampsia, gestational diabetes, increased incidence of mechanical delivery interventions, and stillbirth (4, 5). Also, compared with normal-weight pregnant women, pregnant women with low pre-pregnancy weight are associated with an increased risk of low birth weight (6, 7), fetal growth restriction (FGR) (8, 9), preterm and premature birth (10) and anemia (11). Therefore, this study presumed that a high or low BMI might increase the risk of infant mortality. To this end, a meta-analysis was carried out in this study to systematically assess the association between maternal BMI and the risk of infant mortality.

## Methods

### Search Strategy

This study was conducted according to the guidelines for the Meta-analysis Of Observational Studies in Epidemiology (MOOSE) (12). The electronic databases, including Pubmed, Embase database, and Cochrane Library, were systemically searched by two investigators (Huo and Zhang) from inception to November 26th, 2020, with no language restriction. Three sets of medical subject terms (MeSH) were used to search the studies, including “body mass index,” “mortality,” and “infant.” Additionally, a manual library search was also conducted to ensure a comprehensive search. A detailed search strategy is presented in Appendix 1.

### Study Selection

The study inclusion criteria were as follows: (1) studies focused on maternal BMI; (2) the study outcomes reported the risk of infant, neonatal, post-neonatal or early neonatal mortality; (3) the study type was restricted to cohort study or randomized controlled trial (RCT); (4) the maximum covariates adjusted hazard ratios (HRs), relative risks (RRs), or odds ratios (ORs) were available, or might be obtained through calculation; (5) if a cohort population was investigated repeatedly, studies containing the longest follow-up period or the largest population were included. At the same time, studies conforming to any one of the following criteria were excluded, including: (1) studies not focusing on maternal BMI or those with unavailable maternal BMI data; (2) the study endpoints did not include risk of infant, neonatal, post-neonatal or early neonatal mortalit; (3) case-control or cross-sectional studies; (4) the related HRs, RRs or ORs were not available; (5) data from one population were used repeatedly; (6) case reports, case series, conference abstracts, reviews or letters were also excluded from this study.

### Data Extraction and Quality Assessment

Using a uniform data list, the following data were extracted by two investigators (Huo and Wang), including the first author, published year, country, sample size, study period, parity status, parity, BMI category and outcomes. Any disagreement during the data extraction process was arbitrated by a third investigator (Jia). In addition, the Newcastle-Ottawa Scale (NOS) (13) was also adopted to assess the study quality, with a total score of 9 stars. Studies with a NOS score ≥ 6 stars were considered as high-quality studies, while those with a NOS < 6 stars were considered as low-quality studies.

### Statistical Analysis

Infant death was defined as the death of an infant aged < 1 year. Early neonatal death referred to the death of a newborn before 7 days. Neonatal death was defined as the death of an infant within 28 days of birth. Post-neonatal death was defined as the death of an infant older than 28 days but < 1 year of age.

In this study, the primary endpoint was the qualitative analysis on the relationship between maternal BMI and the risk of infant mortality. To be specific, the impacts of maternal underweight, overweight, and obesity on the corresponding risks of infant, neonatal, post-neonatal and early neonatal mortalities were systemically analyzed through comparing the maternal normal to non-normal weights (such as underweight vs. Normal weight, overweight vs. Normal weight, obesity vs. Normal weight). To use more available data, HRs were roughly equal to RRs in cohort studies (14). In addition, due to the low incidence rates of study outcomes in the total population and subgroup populations (<5%), differences between various measures of relative risk were negligible (such as ORs or RRs) (15). All the pooled data from cohort studies were expressed as RRs. Furthermore, the *I*^2^ statistic was utilized to evaluate the heterogeneity among studies, and *I*^2^ values of 25, 50, and 75% indicated low, moderate, and high inconsistency, respectively. Besides, we performed subgroup and meta-regression analyses to further explore the potential sources of heterogeneity between studies. Moreover, sensitivity analysis, which was performed by excluding one study at a time, was also performed to examine the effect of one study on the pooled results. To more conservatively estimate the pooled RRs, the random-effect model was adopted, since it was able to well-explain the heterogeneity between studies. Besides, Egger's tests were conducted to assess the publication bias (16). If a group of studies contained 2 subgroups (such as obesity 30–39.9 kg/m^2^, ≥40 kg/m^2^), they were considered as 2 studies and analyzed separately.

The secondary endpoint of this study was the quantitative analysis on whether maternal BMI level was associated with the risks of infant, neonatal, post-neonatal and early neonatal mortalities. In parallel, a dose-response was assessed. To this end, we performed a dose-response analysis based on the theory put forward by Xu et al. (17). Specifically, in this “one-stage” framework approach, each included study was considered as a cluster across the entire population, which required that the studies should include at least two categories. The method was adopted to the restricted cubic splines to fit the potential non-linear trends at three nodes, and the non-linear *p*-values were calculated by testing the second spline coefficient to zero. A non-linear model was applied in the case of *p* ≤ 0.05; conversely, a linear-model was used. Generally speaking, when the reference category included in the analysis was not the lowest, we used the Excel macro file produced by Hamling et al. (18) based on the theory proposed by Greenland and Longnecker (19) to convert risk estimates. When the number of cases in a category was not available, we contacted the original authors. Further, the average of the upper and lower bounds was taken as the midpoint for each BMI category, and then the respective RRs were assigned to each midpoint. Meanwhile, when the study interval was open, the amplitude was assumed to be the same as that of the adjacent category (20).

**Figure 1:**
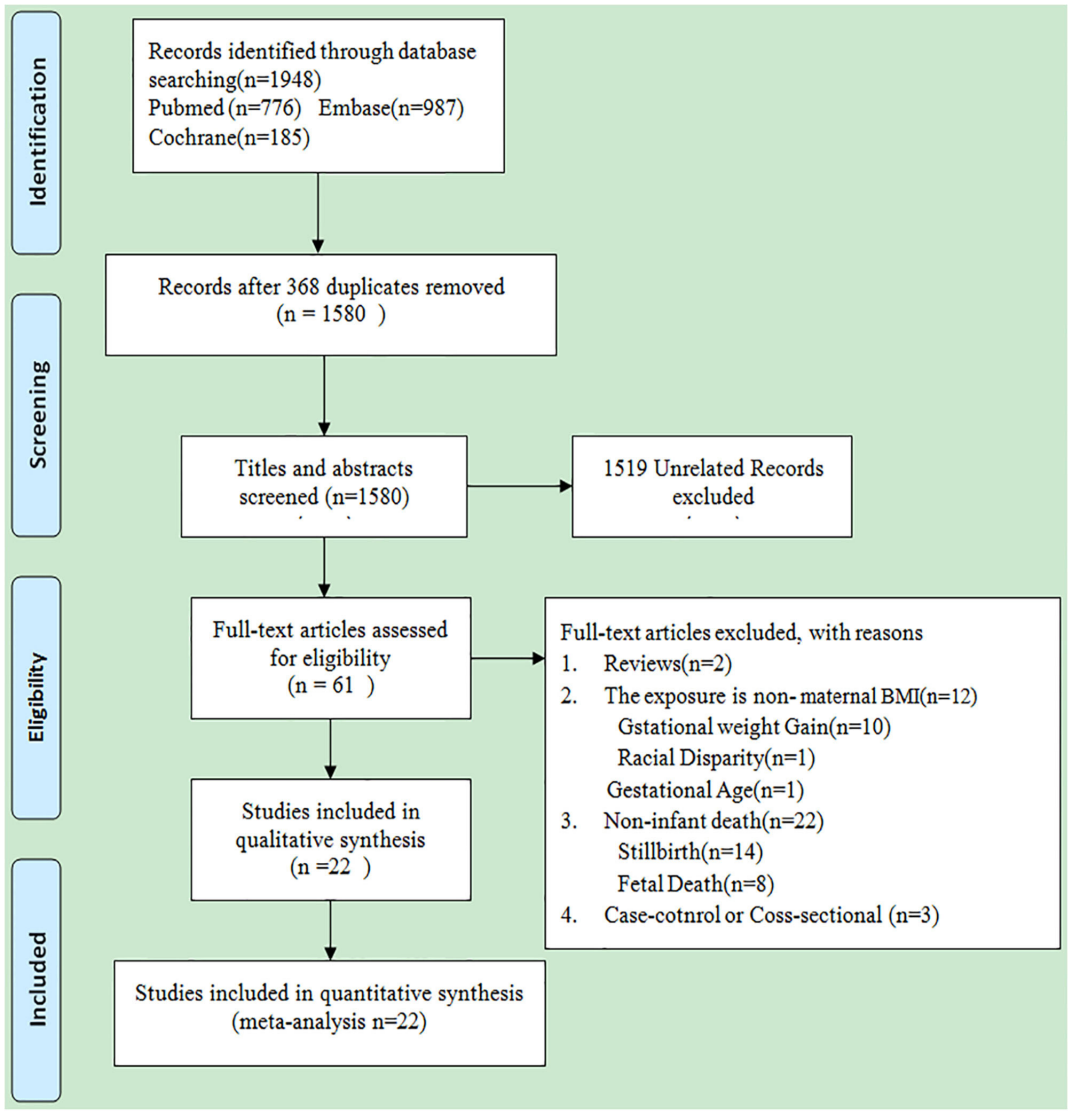
Flow chart of the study retrieval process.

A total of 1,948 studies were searched from 3 electronic databases, including PubMed, Embase, and Cochrane library, as shown in Figure 1. No additional study was identified by manual search. Of these 1,948 studies, 368 were excluded due to duplication; meanwhile, 1,519 irrelevant studies were also removed after screening the titles and abstracts. The full-texts of the remaining 61 studies were carefully read, among which 39 were excluded for the following reasons: (1) review (*n* = 2); (2) the exposure was non-maternal BMI (*n* = 12); (3) non-infant death (*n* = 22); (4) case-control or cross-sectional study (*n* = 3). Finally, 22 cohort studies were included for final analysis (21–42). The detailed characteristics of these studies are shown in Table 1. As shown in Supplementary Table 1, 5 of these 22 studies had the NOS scores of 6 stars; 11 of 7 stars; 5 of 8 stars and 1 of 9 stars.

**Table 1:** The detailed baseline characteristics of the 22 cohort studies.

**References**	**Country**	**Sample Size**	**Study period**	**Parity status**	**Parity**	**Infant death**	**BMI Categories**	**Outcomes**
Kalk et al. (22)	Germay	505	2000.1–2003.12	Mixed	Singleton	22	<18.5 kg/m^2^; 18.5–24.9 kg/m^2^; BMI 25–29.9 kg/m^2^; BMI >30 kg/m^2^	Neonatal death
Baeten et al. (24)	USA	159,072	1992–1996	Primiparous	Singleton	406	<20 kg/m^2^; 20–24.9 kg/m^2^; BMI 25–29.9 kg/m^2^; BMI >30 kg/m^2^	Infant death
Tennant et al. (27)	UK	29,856	2003–2005	Mixed	Singleton	52	<18.5 kg/m^2^; 18.5–24.9 kg/m^2^; BMI 25–29.9 kg/m^2^;BMI >30 kg/m^2^	Neonatal death, infant death, early neonatal death, post-neonatal death
Kristensen et al. (28)	Denmark	24,505	1989–1996	Primiparous multiparous	Singleton	75	<18.5 kg/m^2^; 18.5–24.9 kg/m^2^; BMI 25–29.9 kg/m^2^; BMI >30 kg/m^2^	Neonatal death
Leung et al. (29)	China	29,303	1995–2005	Mixed	Singleton	47	<18.5kg/m^2^; 18.5–24.9 kg/m^2^; BMI 25–29.9 kg/m^2^; BMI >30 kg/m^2^	Neonatal death
Nohr et al. (30)	Denmark	1,199,183	1992–2006	Mixed	Singleton	2,215	<18.5 kg/m^2^; 18.5–24.9 kg/m^2^; BMI 25–29.9 kg/m^2^; BMI 30–34.9 kg/m^2^, BMI>35 kg/m^2^	Neonatal death, infant death, post-neonatal death
Khashan and Kenny (32)	Ireland	99,403	2004–2006	Mixed	Singleton	207	<18.5 kg/m^2^; 18.5–24.9 kg/m^2^; BMI 25–29.9 kg/m^2^; BMI 30–40 kg/m^2^, BMI>40 kg/m^2^	Neonatal death
Nohr et al. (35)	Denmark	85,375	1996–2002	Mixed	Singleton	230	<18.5 kg/m^2^; 18.5–24.9 kg/m^2^; BMI 25–29.9 kg/m^2^; BMI>30 kg/m^2^	Neonatal death
Thompson et al. (36)	USA	166,301	2004.3–2004.12	Mixed	Mixed	1,015	<18.5 kg/m^2^; 18.5–24.9 kg/m^2^; BMI 25–29.9 kg/m^2^; BMI 30–39.9 kg/m^2^, BMI>40 kg/m^2^	Infant death
Salihu et al. (38)	USA	1,405,698	1978–1997	Mixed	Singleton	7,622	18.5–24.9 kg/m^2^, BMI 25–29.9 kg/m^2^; BMI 30–39.9 kg/m^2^, BMI>40 kg/m^2^	Neonatal death
Denison et al. (21)	UK	124,280	2003–2010	Mixed	Singleton	61	<18.5 kg/m^2^; 18.5–25 kg/m^2^; BMI 25–30 kg/m^2^; BMI 30–40 kg/m^2^; BMI>40 kg/m^2^	Neonatal death
Smith et al. (23)	Scotland	187,290	1991–2001	Primiparous multiparous	Singleton	338	<20 kg/m^2^; 20–24.9 kg/m^2^; BMI 25–29.9 kg/m^2^; BMI 30–34.9 kg/m^2^; BMI≥35 kg/m^2^	Neonatal death
Cedergren (25)	Sweden	611,852	1992–2001	Mixed	Singleton	883	19.8–26 kg/m^2^; BMI 29.1–35 kg/m^2^; BMI 35.1–40 kg/m^2^; BMI>40 kg/m^2^	Early neonatal death
Mcintyre et al. (34)	Australia	75,432	1998–2009	Mixed	Singleton	262	<18.5 kg/m^2^; 18.5–25 kg/m^2^; BMI 25–30 kg/m^2^; BMI 30–35 kg/m^2^; BMI 35–40 kg/m^2^ BMI≥40 kg/m^2^	Neonatal death
Wallace et al. (40)	UK	55,105	1976–2007	Mixed	Singleton	175	<18.5 kg/m^2^; 18.6–24.9 kg/m^2^; BMI 25–29.9 kg/m^2^; BMI 30–34.9 kg/m^2^; BMI≥35 kg/m^2^	Neonatal death
Declercq et al. (26)	USA	6,419,836	2012–2013	Mixed	Singleton	36,691	<18.5 kg/m^2^; 18.6–24.9 kg/m^2^; BMI 25–29.9 kg/m^2^; BMI 30–34.9 kg/m^2^; BMI 35–39.9 kg/m^2^; BMI≥40 kg/m^2^	Infant death
Johansson et al. (31)	USA	1,857,822	1992–2010	Mixed	Singleton	5,642	<18.5 kg/m^2^; 18.6–24.9 kg/m^2^; BMI 25–29.9 kg/m^2^; BMI 30–34.9 kg/m^2^; BMI≥35 kg/m^2^	Neonatal death, infant death, post-neonatal death
Yu et al. (41)	USA	212,889	2003–2013	Multiparous	Singleton	1,002	<18.5 kg/m^2^; 18.5–24.9 kg/m^2^; BMI 25–29.9 kg/m^2^; BMI>30 kg/m^2^	Neonatal death, infant death, post-neonatal death
Madi et al. (33)	Brazil	3,892	1998–2010	Mixed	NA	69	18.5–24.9 kg/m^2^; BMI≥30 kg/m^2^	Early neonatal death
Rai et al. (37)	India	55,629	NA	Mixed	Singleton	840	<18.5 kg/m^2^; 18.5–22.9 kg/m^2^; BMI 23–27.4 kg/m^2^; BMI≥27.5 kg/m^2^	Neonatal death, early neonatal death
Vincent et al. (39)	Canada	717,080	2009–2013	Mixed	NA	3,241	18.6–24.9 kg/m^2^; BMI 25–29.9 kg/m^2^; BMI 30–34.9 kg/m^2^; BMI 35–39.9 kg/m^2^; BMI≥40 kg/m^2^	Infant death
Melchor et al. (42)	Spain	11,985	2013–2017	Mixed	Singleton	18	18.6–24.9 kg/m^2^; BMI>30 kg/m^2^	Neonatal death

*BMI, body bass index; NA, not applicable. underweight, <18.5 kg/m^2^; normal weight, 18.5–24.9 kg/m^2^; overweight, 25–29.9 kg/m^2^; Obese, ≥30 kg/m^2^*.

## Meta-Analysis

### Infant Mortality

**Figure 2:**
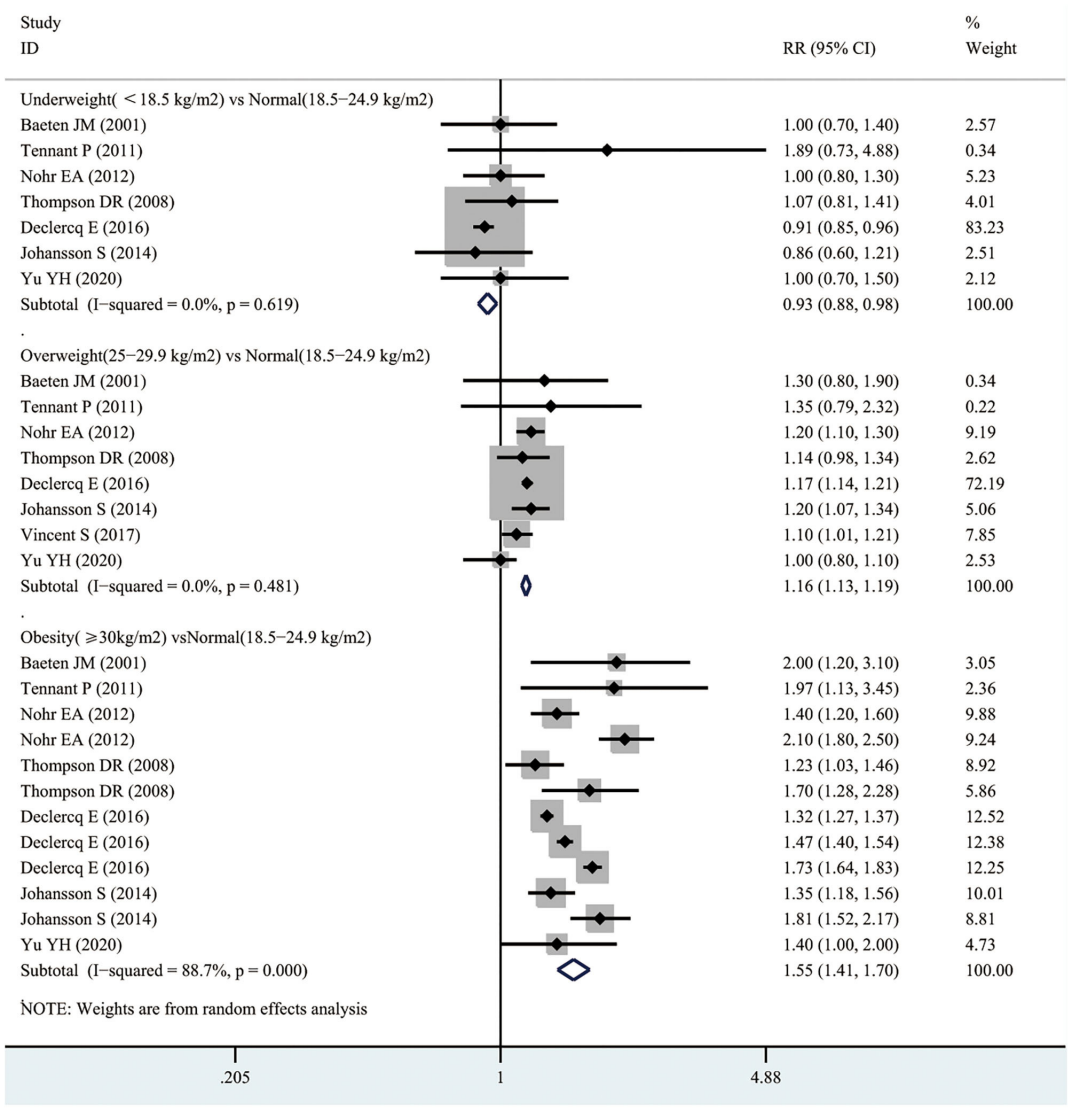
Forest plots of maternal BMI and the risk of infant mortality.

As shown in Figure 2, 7 studies recruiting 10,044,959 participants reported the association between maternal underweight and the risk of infant mortality. Compared with normal-weight pregnant women, underweight pregnant women appeared to have a reduced risk of infant mortality (RR 0.93, 95% CI 0.88–0.98; *I*^2^ 0%). However, 8 studies including 10,762,039 participants showed that the risk of infant mortality increased by 16% in overweight pregnant women compared with normal-weight pregnant women (RR 1.16, 95% CI 1.13–1.19; *I*^2^ 0%). Similarly, obese pregnant women had a 55% higher risk of infant mortality than normal weight pregnant women (RR 1.55, 95% CI 1.41–1.70; *I*^2^ 88.7%). Furthermore, we assessed the publication bias of maternal BMI and the risk of infant mortality by performing funnel plots. Subjectively, the funnel plot appeared to be symmetric, as shown in Supplementary Figure 1A. There was no obvious evidence of publication bias upon Egger's test (*p* = 0.773).

**Figure 3:**
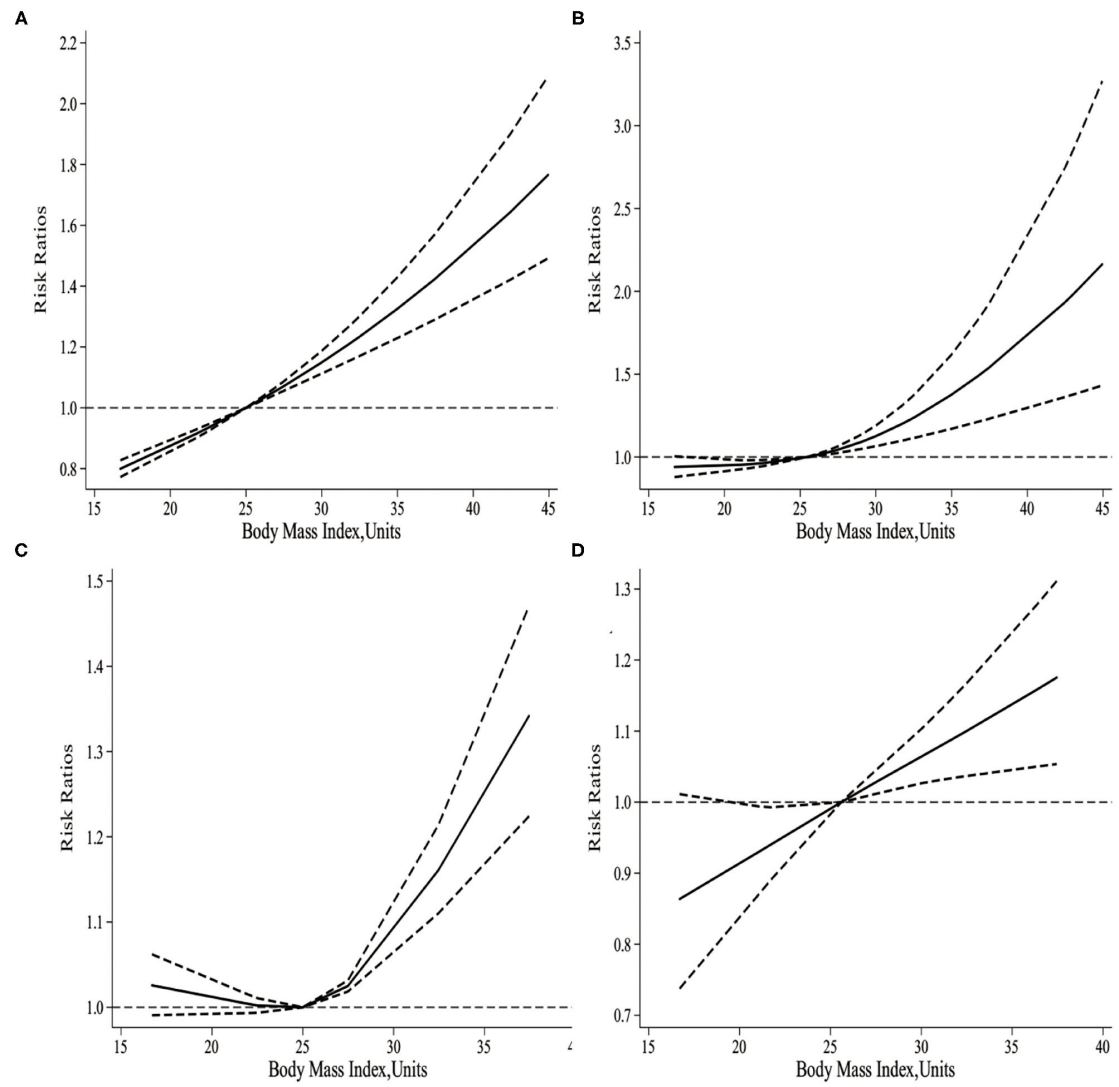
**(A)** The dose-response of maternal BMI and the risk of infant mortality. **(B)** The dose-response of maternal BMI and the risk of neonatal mortality. **(C)** The dose-response of maternal BMI and the risk of post-neonatal mortality. **(D)** The dose-response of maternal BMI and the risk of early neonatal mortality.

As displayed in Figure 3A, there was a non-linear relationship between maternal BMI and the risk of infant mortality. Specifically, the dose-response analysis on 8 studies showed that maternal BMI level was non-linearly and positively associated with the risk of infant mortality (*p*_*nonlinearity*_ < 0.001). The risk of infant mortality decreased with the increase in BMI level from 16.7 to 25 kg/m^2^, but with a progressively increasing trend. Typically, the risk of infant mortality increased significantly when maternal BMI was > 25 kg/m^2^.

### Neonatal Mortality

**Figure 4:**
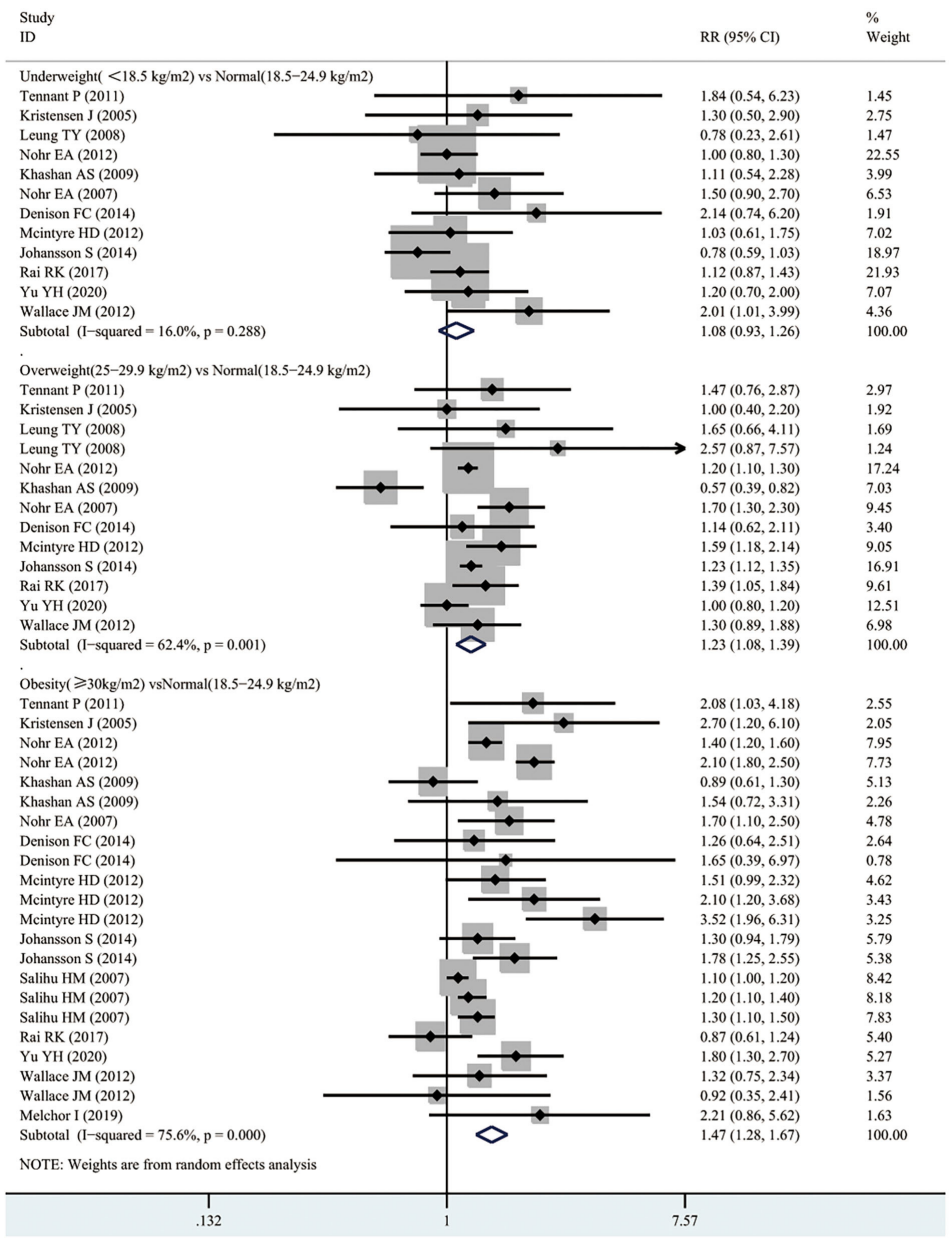
Forest plots of maternal BMI and the risk of neonatal mortality.

As presented in Figure 4, 12 studies involving 3,848,782 participants investigated the relationship between maternal underweight and the risk of neonatal mortality. According to the pooled results, underweight pregnant women did not show significantly increased risk of infant mortality compared with normal weight pregnant women (RR 1.08, 95% CI 0.93–1.26; *I*^2^ 16%). However, the pooled results from 12 studies including 3,848,782 participants indicated that overweight pregnant women contributed to a 23% higher risk of neonatal mortality than normal weight pregnant women (RR 1.23, 95% CI 1.08–1.39; *I*^2^ 62.4%). Similarly, obese pregnant women showed a 47% increased risk of neonatal mortality compared with normal weight pregnant women (RR 1.55, 95% CI 1.28–1.67; *I*^2^ 75.6%). Moreover, funnel plot was conducted to assess the publication bias of maternal BMI and the risk of neonatal mortality. Subjectively, the funnel plot was symmetrical, as presented in Supplementary Figure 1B. No obvious evidence of publication bias was found by Egger's test (*p* = 0.099).

It was illustrated from the dose-response analysis on 15 studies in Figure 3B that, there was a non-linear relationship between maternal BMI and the risk of neonatal mortality. Specifically, maternal BMI level was non-linearly and positively associated with the risk of neonatal mortality (*p*_*nonlinearity*_= 0.002). Besides, the risk of neonatal mortality increased slowly with the maternal BMI level increasing from 16.3 to 25 kg/m^2^. However, the risk of neonatal mortality significantly increased when the maternal BMI was > 25 kg/m^2^.

### Post-neonatal Mortality

**Figure 5:**
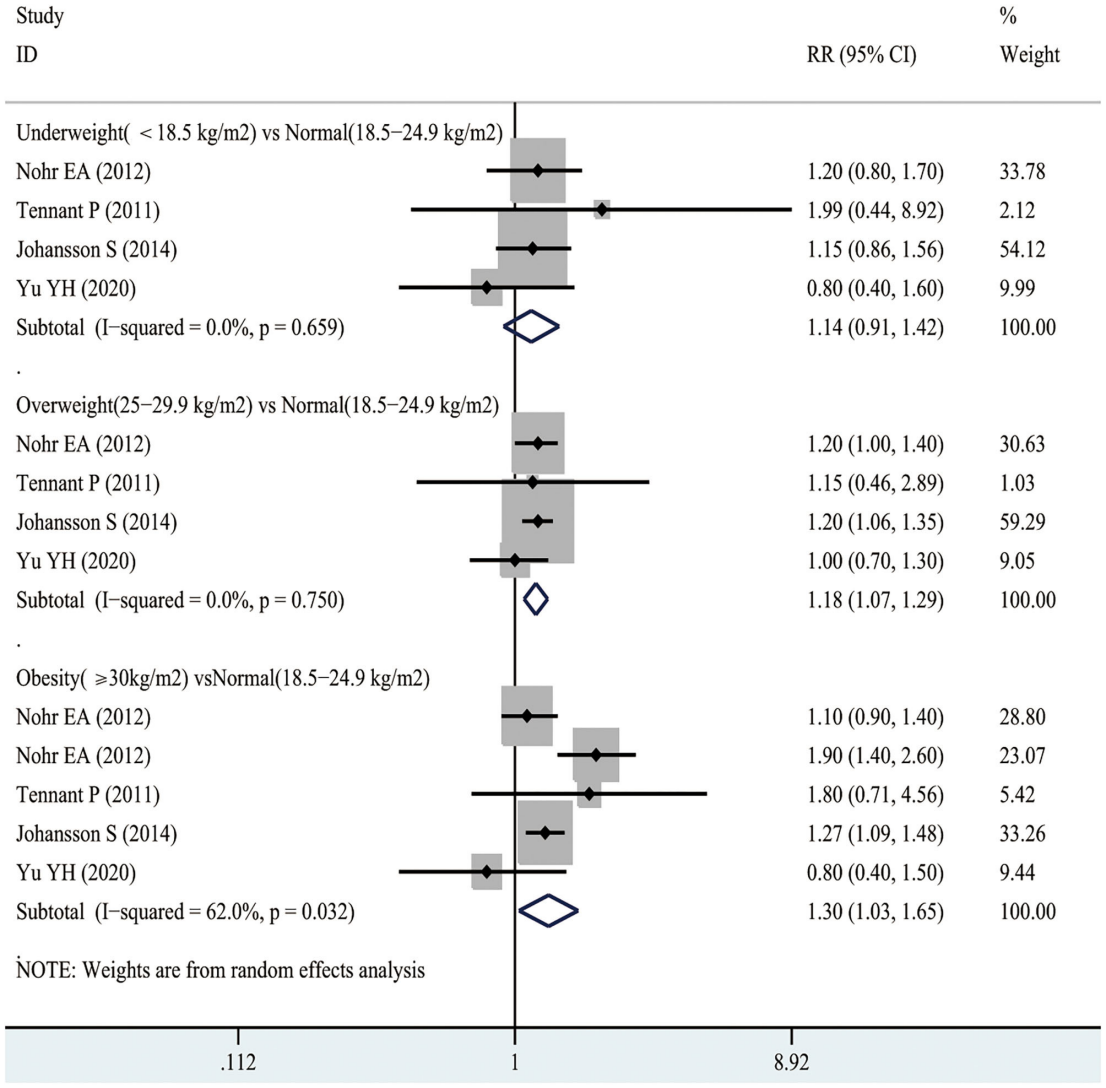
Forest plots of maternal BMI and the risk of post-neonatal mortality.

According to Figure 5, 4 studies involving 3,299,750 participants reported the association between maternal underweight and the risk of post-neonatal mortality. As a result, maternal underweight might not lead to an increased risk of infant mortality relative to normal weight pregnant women (RR 1.14, 95% CI 0.91–1.42; *I*^2^ 0%). However, 4 studies including 3,299,750 participants showed an 18% increased risk of post-neonatal mortality in overweight pregnant women compared with normal-weight pregnant women (RR 1.18, 95% CI 1.07–1.29; *I*^2^ 0%). Similarly, compared with normal weight pregnant women, the risk of post-neonatal mortality in obese pregnant women increased by 30% (RR 1.30, 95% CI 1.03–1.65; *I*^2^ 62%). The publication bias of maternal BMI and the risk of post-neonatal mortality was assessed through funnel plot analysis. It was observed from Supplementary Figure 1C that, the funnel plot appeared to be asymmetric, while there was no evidence of publication bias by Egger's test (*p* = 0.928).

As shown in Figure 3C, there was a non-linear relationship between maternal BMI and the risk of post-neonatal mortality. Specifically, the dose-response analysis on 4 studies revealed a non-linear association between maternal BMI level and the risk of post-neonatal mortality (*p*_*nonlinearity*_= 0.011). With the increase in maternal BMI level from 16.7 to 25 kg/m^2^, the risk of post-neonatal mortality did not increase, but there was a gradually increasing trend. When the maternal BMI level was > 25 kg/m^2^, the risk of post-neonatal mortality increased significantly.

### Early Neonatal Mortality

**Figure 6:**
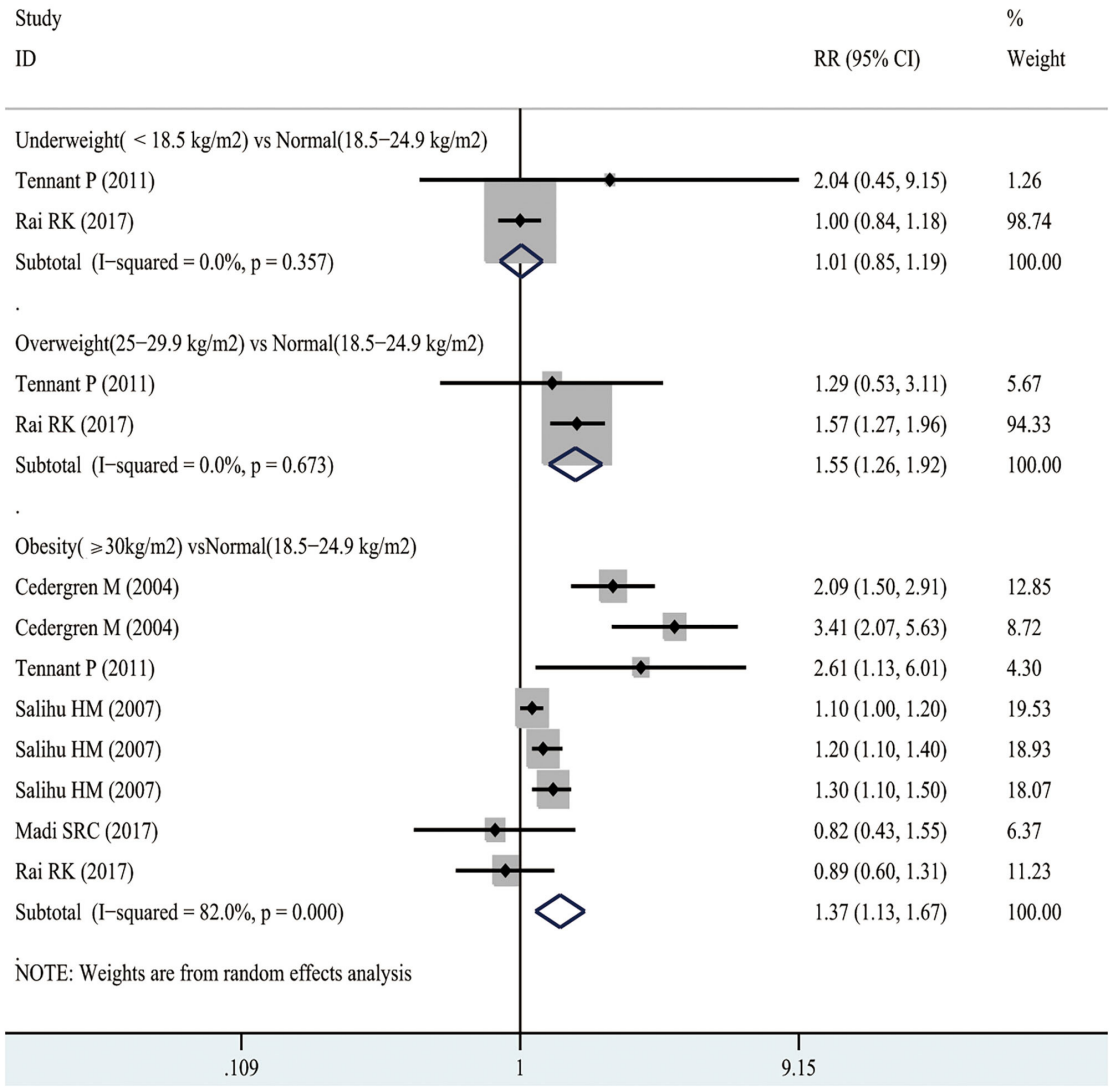
Forest plots of maternal BMI and the risk of early neonatal mortality.

According to Figure 6, 2 studies involving 641,708 participants investigated the association between maternal underweight and the risk of early neonatal mortality. As suggested by our results, maternal underweight might not increase the risk of early neonatal mortality compared with normal weight pregnant women (RR 1.01, 95% CI 0.85–1.19; *I*^2^ 0%). However, 2 studies recruiting 641,708 participants suggested that maternal overweight increased the risk of post-neonatal mortality compared with normal-weight pregnant women (RR 1.55, 95% CI 1.26–1.92; *I*^2^ 0%). Similarly, maternal obesity also increased the risk of early neonatal mortality (RR 1.37, 95% CI 1.13–1.67; *I*^2^ 82%). Moreover, funnel plot was conducted to evaluate the publication bias of maternal BMI and the risk of early neonatal mortality, as shown in Supplementary Figure 1D. No obvious evidence of publication bias was observed by Egger's test (*p* = 0.128).

As observed from Figure 3D, there was a non-linear relationship between maternal BMI and the risk of early neonatal mortality. Specifically, the dose-response analysis on 5 studies showed that maternal BMI level was linearly related to the risk of early neonatal mortality (*p*_*nonlinearity*_ = 0.0625). With the increase in maternal BMI level from 16.7 to 25.6 kg/m^2^, the risk of early neonatal mortality showed an increasing trend; meanwhile, when the maternal BMI level was > 25.6 kg/m^2^, the risk of early neonatal mortality increased significantly.

### Subgroup Analyses and Sensitivity Analyses

Subgroup analyses were performed to explore the potential sources of heterogeneity under these clinical characteristics, including country, NOS scores, infant death cases, and published year. Also, meta-regression was performed to further clarify the potential sources of heterogeneity. In subgroup analysis on infant mortality (obesity vs. normal), the studies reporting infant death cases > 500 and those published after 2000 might partially contribute to the heterogeneity, while meta-regression did not identify any potential source of heterogeneity. In the subgroup analysis on neonatal mortality (overweight vs. normal), studies from Europe and those published before 2000 might partially contribute to the heterogeneity. Similarly, meta-regression did not identify any source of potential heterogeneity. In subgroup analysis on neonatal mortality (obesity vs. normal), no potential source of heterogeneity was identified, and yet meta-regression suggested that the infant death cases (*p* = 0.06) might partially contribute to heterogeneity. Finally, in the subgroup analysis on post-neonatal mortality (obesity vs. normal) and early neonatal mortality (obesity vs. normal), studies with NOS scores > 8-9 might contribute to the heterogeneity of post-neonatal mortality, whereas no other potential source of heterogeneity was identified. Due to the small number of studies, meta-regression analysis was not performed.

In sensitivity analyses, 1 study was excluded from each analysis at a time, and most results appeared to be robust to the effects of individual studies, as shown in Supplementary Figures 2–4.

## Discussion

As shown by the 22 cohort studies including 13,532,293 participants enrolled in this paper, infant mortality increased significantly among overweight or obese pregnant women, compared with normal pregnant women. Simultaneously, maternal underweight might not increase the risk of infant mortality compared with normal-BMI pregnant women, but a trend toward a lower risk of infant mortality was observed.

Two meta-analyses in 2014 systematically analyzed the relationship between maternal BMI level and the risk of infant mortality. For instance, Meehan et al. restricted the study population to obese pregnant women and their results showed that maternal obesity was significantly associated with an increased risk of infant mortality (43). In addition, Aune et al. suggested that even the modest increases in maternal BMI were strongly associated with an increase in infant mortality, while the dose-response analysis showed that low maternal underweight did not significantly increase the risk of infant mortality (44), but only four cohort studies were included. Results obtained from this paper enriched previous studies.

In general, the causes of maternal obesity are complex and multifactorial, and the potential increased risk induced by these causes may be related to obesity itself or the presence of comorbidities such as gestational diabetes and hypertensive disorders (45). A recent meta-analysis shows that maternal overweight or obesity is significantly associated with increased risks of stillbirth, macrosomia, admission to the neonatal intensive care unit (NICU) and large for gestational age (LBW), while maternal underweight is related to the increased risks of preterm birth, small for gestational age (SGA) and LBW (46). Compared with maternal underweight, maternal overweight or obesity appears to be associated with more adverse effects on infants. Besides, it has been shown that even in the absence of clinical disease, obese women have increased inflammatory response (47), vascular and endothelial dysfunction, and lipid metabolic disorders, leading to hyperlipidemia. Hyperlipidemia will cause reduced prostacyclin secretion and increased thromboxane secretion (48), which thus increases the risk of placental thrombosis, reduces placental perfusion (47), elevates the risks of placental infarction and abruption in late pregnancy, leading to preterm birth (49, 50). Also, preterm delivery is found to be closely related to the development of respiratory distress syndrome, which may be the important cause of preterm death (34). On the other hand, most studies have focused on overweight or obese pregnant women, while relatively few studies are conducted on underweight pregnant women.

In the analysis of infant mortality (underweight vs. normal), the risk of infant mortality appeared to be reduced in underweight pregnant women, which seemed to be counter-intuitive to popular convictions. However, in sensitivity analysis, when the study by Declercq et al. was removed, the pooled results were insignificant (RR 1.01, 95% CI 0.88–1.15). Besides, the study by Declercq et al. (26) showed that those underweight pregnant women were linked with a reduced risk of infant mortality compared with normal-weight women, but the primary endpoint of this study was the effect of obesity on infant mortality, and no plausible explanation was given for this results. It has been suggested that women with lower BMI levels can better recognize the reduced fetal movement and therefore take the necessary early interventions, which may partially explain the possible mechanism underlying the risk of infant mortality in underweight pregnant women. In addition, the theory for maternal underweight being protective could also involve mechanisms of cellular hibernation and conservation in the face of stress that may result in earlier maturation of fetal pathways conferring improved survivability. Further research with more prospective studies is needed in the future. Besides, the relationships between maternal BMI level and infant mortality may also be affected by factors such as economic conditions, nutritional status and race. For example, Salihu et al. conducted a restricted analysis on neonatal mortality using data from Missouri in 1978–1997 and found that BMI was associated with the risk of neonatal mortality among white pregnant women, whereas black mothers were related to an overall risk of infant mortality (38).

This study has the following strengths. Firstly, all studies included in this paper were cohort studies with strong levels of evidence, most of them were population-based studies with large samples, and all studies were of high quality. Secondly, this paper systematically conducted qualitative and dose-response analyses to validate the before-and-after results. Findings in this paper also complemented and updated previous studies. Thirdly, sensitivity analysis, subgroup analysis, and meta-regression analysis were simultaneously conducted to maximally search the potential sources of heterogeneity.

Meanwhile, the following limitations should be noted in this work. Firstly, most of the included studies adopted self-reporting for the assessment of BMI, which might produce a certain bias, and for some groups of overweight women, this bias might lead to an underestimation of the measure of effect. Secondly, there were great heterogeneities among some of the studies. Although subgroup and meta-regression analyses were performed, the sources of heterogeneity were not well-explained yet. Thirdly, the maternal BMI level was affected by many factors such as age, economic conditions and nutritional status. Although the extracted RRs were adjusted for maximum covariates, it is still unknown about the potential impacts of other factors on the results. Last but not least, an important limitation was lack of birthweight data that may shed more insight into survival.

## Conclusions

Maternal overweight or obesity significantly increases the risks of infant mortality, neonatal mortality, early neonatal mortality, and post-neonatal mortality compared with normal BMI in a dose-dependent manner. Also, maternal underweight does not increase the risk of infant mortality, neonatal mortality, early neonatal mortality, or post-neonatal mortality; instead, it tends to decrease the risk of infant mortality. Early weight management may provide potential benefits to infants, and more prospective studies with large samples are warranted to verify this finding in the future.

## Data Availability Statement

The raw data supporting the conclusions of this article will be made available by the authors, without undue reservation.

## Author Contributions

NH participated in the data collection, data review, relevant data extraction, data analysis, statistical analysis, and the writing of the manuscript. KZ, LinW, GJ, and LiW participated in checking data extraction as well as in the data analysis, statistical analysis, and the writing of the manuscript. WL participated in checking data extraction as well as in the statistical analysis. WC participated in performing the risk-of-bias assessment. All authors saw and approved the final version.

## Conflict of Interest

The authors declare that the research was conducted in the absence of any commercial or financial relationships that could be construed as a potential conflict of interest.

## Supplementary Material

Supplementary Figure 1 **(A)** The funnel plot of maternal BMI and the risk of infant mortality. **(B)** The funnel plot of maternal BMI and the risk of neonatal mortality. **(C)** The funnel plot of maternal BMI and the risk of post-neonatal mortality. **(D)** The funnel plot of maternal BMI and the risk of early neonatal mortality.

Supplementary Figure 2 **(A)** The sensitivity analysis of maternal underweight and the risk of infant mortality. **(B)** The sensitivity analysis of maternal overweight and the risk of infant mortality. **(C)** The sensitivity analysis of maternal obesity and the risk of infant mortality.

Supplementary Figure 3 **(A)** The sensitivity analysis of maternal underweight and the risk of neonatal mortality. **(B)** The sensitivity analysis of maternal overweight and the risk of neonatal mortality. **(C)** The sensitivity analysis of maternal obesity and the risk of neonatal mortality.

Supplementary Figure 4 **(A)** The sensitivity analysis of maternal underweight and the risk of post-neonatal mortality. **(B)** The sensitivity analysis of maternal overweight and the risk of post-neonatal mortality. **(C)** The sensitivity analysis of maternal obesity and the risk of post-neonatal mortality. **(D)** The sensitivity analysis of maternal obesity and the risk of early neonatal mortality.
