# Supplementary material for: Association of Maternal Body Mass Index With Risk of Infant Mortality: A Dose-Response Meta-Analysis
Source: Front Pediatr. 2021 Mar 12;9:650413. doi: 10.3389/fped.2021.650413 (PMC7994890; doi:10.3389/fped.2021.650413)
Supplement: Supplementary file 5 [file Table_1.docx]

| ***Supplementary Table 1*. Quality Assessment of the 22 Cohort Studies** | | | | | | | | |  |
| --- | --- | --- | --- | --- | --- | --- | --- | --- | --- |
| **Study**  **(First Author, Year)** | **Selection** | | | | **Comparability** | **Outcome** | | | **Total** |
|  | **Exposed**  **Cohort** | **Nonexposed**  **Cohort** | **Ascertainment**  **of Exposure** | **Outcome**  **of Interest** |  | **Assessment**  **of Outcome** | **Length**  **Of Follow-up** | **Adequacy**  **of Follow-up** |  |
| Kalk P,2009 | 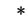 | 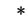 |  | 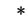 | 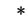 | 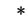 | 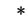 |  | **6** |
| Baeten JM,2001 | 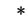 | 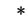 | 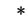 | 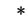 | 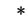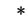 | 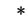 | 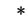 |  | **8** |
| Tennant P,2011 | 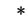 | 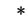 |  | 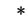 | 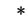 | 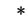 | 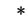 |  | **7** |
| Kristensen J,2005 | 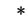 | 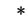 | 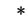 | 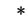 | 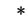 |  | 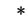 | 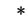 | **7** |
| Leung TY,2008 | 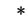 | 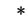 | 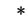 | 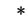 | 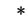 | 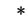 | 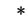 | 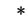 | **8** |
| Nohr EA,2012 | 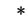 | 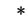 | 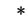 | 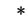 | 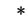 | 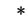 | 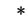 | 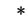 | **8** |
| Khashan AS,2009 | 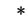 |  | 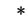 | 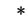 | 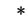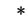 | 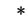 | 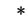 |  | **7** |
| Nohr EA,2007 | 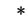 | 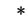 | 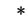 | 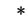 | 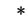 | 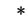 | 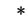 | 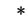 | **8** |
| Thompson DR,2008 | 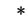 | 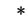 | 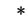 |  | 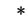 | 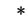 | 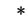 | 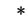 | **7** |
| Salihu HM,2007 | 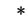 | 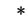 | 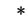 | 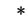 | 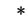 |  | 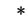 | 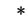 | **7** |
| Denison FC,2014 | 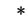 | 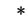 | 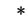 | 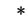 | 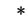 |  | 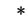 | 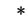 | **7** |
| Smith G.2007 | 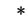 | 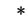 |  | 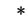 | 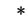 |  | 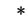 | 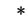 | **6** |
| Cedergren M,2004 | 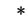 | 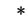 |  | 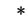 | 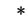 |  | 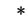 |  | **6** |
| Mcintyre HD,2012 | 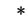 | 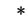 |  | 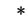 | 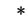 |  | 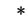 | 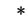 | **6** |
| Wallace JM,2012 | 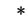 | 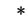 | 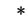 | 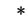 |  |  |  |  | **8** |
| Declercq E,2016 |  |  |  |  |  |  |  |  | **7** |
| Johansson S,2014 |  |  |  |  |  |  |  |  | **7** |
| Yu YH,2020 |  |  |  |  |  |  |  |  | **9** |
| Madi SRC,2017 |  |  |  |  |  |  |  |  | **7** |
| Rai RK,2017 |  |  |  |  |  |  |  |  | **6** |
| Vincent S,2017 |  |  |  |  |  |  |  |  | **7** |
| Melchor I,2019 |  |  |  |  |  |  |  |  | **7** |
