# Supplementary material for: Association of Maternal Body Mass Index With Risk of Infant Mortality: A Dose-Response Meta-Analysis
Source: Front Pediatr. 2021 Mar 12;9:650413. doi: 10.3389/fped.2021.650413 (PMC7994890; doi:10.3389/fped.2021.650413)
Supplement: Supplementary file 6 [file Table_2.docx]

| **Supplementary Table 2.Subgroup Analyses and Meta-regression fore Infant Mortality** | | | | | | | | | | | | | | | |
| --- | --- | --- | --- | --- | --- | --- | --- | --- | --- | --- | --- | --- | --- | --- | --- |
|  | **Infant Mortality**  **Obesity(**≥30kg/m^2^**) vs Normal(**18.5-24.9 kg/m^2^**)** | | | | | **Neonatal Mortality**  **Overweight (**25-29.9 kg/m^2^**) vs Normal(**18.5-24.9 kg/m^2^**)** | | | | | | **Neonatal Mortality**  **Obesity(**≥30kg/m^2^**) vs Normal(**18.5-24.9 kg/m^2^**)** | | | |
| **Type** | n | RR,95％CI,I^2^ | *p*^a^ | *p*^b^ | | n | RR,95％CI,I^2^ | | *p*^a^ | | *p*^b^ | n | RR,95％CI,I^2^ | *p*^a^ | *p*^b^ |
| **Country** | 7 |  |  |  | | 12 |  | |  | |  | 13 |  |  |  |
| Others | 0 |  |  |  | | 2 | 1.48(1.21-1.82)I^2^0％ | | 0.519 | | 0.451 | 2 | 1.71(0.95-1.82)I^2^83.8％ | ＜0.001 | 0.765 |
| America | 5 | 1.31(1.18-1.45)I^2^89.6％ | ＜0.001 | 0.301 | | 3 | 1.16(1.00-1.35)I^2^43.2％ | | 0.172 | |  | 4 | 1.28(1.14-1.43)I^2^52％ | 0.042 |  |
| Europe | 2 | 1.39(1.00-1.95)I^2^85.6％ | 0.001 |  | | 7 | 1.21(0.91-1.60)I^2^70.9％ | | 0.001 | |  | 7 | 1.60(1.27-2.01)I^2^65.6％ | 0.002 |  |
| **NOS Scores** |  |  |  |  | |  |  | |  | |  |  |  |  |  |
| 8-9 | 3 | 1.30(1.98-1.73)I^2^82.7％ | 0.001 | 0.373 | | 4 | 1.39(1.13-1.72)I^2^46.4％ | | 0.113 | | 0.213 | 3 | 1.59(1.22-2.07)I^2^73.9％ | 0.004 | 0.563 |
| 6-7 | 4 | 1.33(1.20-1.47)I^2^90.6％ | ＜0.001 |  | | 8 | 1.14(0.93-1.38)I^2^70.5％ | | 0.001 | |  | 10 | 1.40(1.22-1.6)I^2^65.1％ | ＜0.001 |  |
| **Infant Death** |  |  |  |  | |  |  | |  | |  |  |  |  |  |
| ＜500 | 2 | 1.17(0.82-1.68)I^2^0％ | 0.872 | 0.567 | | 9 | 1.26(1.02-1.56)I^2^67.2％ | | 0.001 | | 0.783 | 9 | 1.65(1.37-2.00)I^2^60.7％ | 0.001 | 0.06 |
| ≥500 | 5 | 1.30(1.18-1.43)I^2^90.0％ | ＜0.001 |  | | 3 | 1.19(1.01-1.39)I^2^54.8％ | | 0.109 | |  | 4 | 1.25(1.10-1.42)I^2^65.2％ | 0.008 |  |
| **Published Year** |  |  |  |  | |  |  | |  | |  |  |  |  |  |
| ＜2000 | 3 | 1.10(0.95-1.27)I^2^0％ | 0.42 | 0.798 | | 4 | 1.25(0.67-2.32)I^2^83.1％ | | ＜0.001 | | 0.674 | 4 | 1.22 (1.08-1.39)I^2^55.4％ | 0.036 | 0.107 |
| ≥2000 | 4 | 1.38(1.25-1.52)I^2^90.0％ | ＜0.001 |  | | 8 | 1.22(1.14-1.31)I^2^14.6％ | | 0.315 | |  | 9 | 1.60(1.35-1.91)I^2^63.8％ | ＜0.001 |  |
|  | **Post-neonatal Mortality**  **Obesity(**≥30kg/m^2^**) vs Normal(**18.5-24.9 kg/m^2^**)** | | | **Early Neonatal Mortality**  **Obesity(**≥30kg/m^2^**) vs Normal(**18.5-24.9 kg/m^2^**)** | | | | | |  |  |  |  |  |  |
| **Type** | n | RR,95％CI,I^2^ | *p*^a^ | n | RR,95％CI,I^2^ | | | *p*^a^ | |  |  |  |  |  |  |
| Country | 4 |  |  | 5 |  | | |  | |  |  |  |  |  |  |
| Others | 0 |  |  | 1 | - | | |  | |  |  |  |  |  |  |
| America | 4 | 1.30(1.03-1.65)I^2^62％ | 0.032 | 3 | 1.19(1.06-1.35)I^2^52.4％ | | | 0.078 | |  |  |  |  |  |  |
| Europe | 0 |  |  | 1 | - | | |  | |  |  |  |  |  |  |
| **NOS Scores** |  |  |  |  |  | | |  | |  |  |  |  |  |  |
| 8-9 | 1 | - |  |  |  | | |  | |  |  |  |  |  |  |
| 6-7 | 3 | 1.22(0.95-1.58)I^2^16.1％ | 0.304 | 5 | 1.37(1.13-1.67)I^2^82％ | | | ＜0.001 | |  |  |  |  |  |  |
| **Infant Death** |  |  |  |  |  | | |  | |  |  |  |  |  |  |
| ＜500 | 1 | - |  | 2 | 1.42(0.46-4.4)I^2^78.5％ | | | 0.031 | |  |  |  |  |  |  |
| ≥500 | 3 | 1.28(1.00-1.64)I^2^90.0％ | 0.018 | 3 | 1.38 (1.13-1.69)I^2^85.4％ | | | ＜0.001 | |  |  |  |  |  |  |
| **Publish Year** |  |  |  |  |  | | |  | |  |  |  |  |  |  |
| ＜2010 | 0 |  |  | 2 | 1.47(1.18-1.82)I^2^87.5％ | | | ＜0.001 | |  |  |  |  |  |  |
| ≥2010 | 4 | 1.30(1.03-1.65)I^2^62％ | 0.032 | 3 | 1.14 (0.63-2.06)I^2^65.3％ | | | 0.056 | |  |  |  |  |  |  |

*p*^a^ for heterogeneity within each subgroup. *p*^b^ for heterogeneity between subgroups with meta-regression analysis. normal weight,18.5-24.9 kg/m^2^.overweight ,25-29.9 kg/m^2^. Obese,≥30kg/m^2^.
