# Supplementary material for: Association of Maternal Body Mass Index With Risk of Infant Mortality: A Dose-Response Meta-Analysis
Source: Front Pediatr. 2021 Mar 12;9:650413. doi: 10.3389/fped.2021.650413 (PMC7994890; doi:10.3389/fped.2021.650413)
Supplement: Supplementary file 7 [file Data_Sheet_1.docx]

Appendix

Pubmed

1. Search: (((((Index, Body Mass) OR (Quetelet Index)) OR (Index, Quetelet)) OR (Quetelet's Index)) OR (Quetelets Index)) OR ("Body Mass Index"[Mesh])

2. Search: (((((((((((((((((((((((((((((((((((((("Mortality"[Mesh]) OR (Mortalities)) OR (Case Fatality Rate)) OR (Case Fatality Rates)) OR (Rate, Case Fatality)) OR (Rates, Case Fatality)) OR (CFR Case Fatality Rate)) OR (Crude Death Rate)) OR (Crude Death Rates)) OR (Death Rate, Crude)) OR (Rate, Crude Death)) OR (Crude Mortality Rate)) OR (Crude Mortality Rates)) OR (Mortality Rate, Crude)) OR (Rate, Crude Mortality)) OR (Death Rate)) OR (Death Rates)) OR (Rate, Death)) OR (Mortality Rate)) OR (Mortality Rates)) OR (Rate, Mortality)) OR (Mortality, Excess)) OR (Excess Mortality)) OR (Excess Mortalities)) OR (Decline, Mortality)) OR (Mortality Declines)) OR (Mortality Decline)) OR (Mortality Determinants)) OR (Determinants, Mortality)) OR (Determinant, Mortality)) OR (Mortality Determinant)) OR (Mortality, Differential)) OR (Differential Mortality)) OR (Differential Mortalities)) OR (Age-Specific Death Rate)) OR (Age-Specific Death Rates)) OR (Death Rate, Age-Specific)) OR (Rate, Age-Specific Death)) OR (Age Specific Death Rate)

3. Search: ("Infant"[Mesh]) OR (Infants)

4 1 and 2 and 3 776

Embase

1. 'body mass'/exp

2. bmi AND body AND mass AND index OR (body AND ban AND mass) OR (body AND mass AND index) OR (quetelet AND index)

3. 1 or 2

4. 'mortality'/exp

5. mortality OR (mortality AND model) OR mortalities

6. #4 OR #5

7. 'infant'/exp

8. infants

9. #7 OR #8

10. #3 AND #6 AND #9 987

Cochrane

1. MeSH descriptor: [Body Mass Index] explode all trees

2. (Quetelet's Index) OR (Quetelet Index) OR (Index, Quetelet) OR (Quetelets Index) OR (Index, Body Mass)

3.1 or 2

4. MeSH descriptor: [Mortality] explode all trees

5. (Mortality Declines) OR (Declines, Mortality) OR (Decline, Mortality) OR (Mortality Decline) OR (Age-Specific Death Rate)

6. (Death Rate, Age-Specific) OR (Age-Specific Death Rates) OR (Rates, Age-Specific Death) OR (Death Rates, Age-Specific) OR (Age Specific Death Rate)

7. (Rate, Age-Specific Death) OR (Case Fatality Rate) OR (Case Fatality Rates) OR (Rates, Case Fatality) OR (Rate, Case Fatality)

8. (Determinant, Mortality) OR (Determinants, Mortality) OR (Mortality Determinants) OR (Mortality, Excess) OR (Excess Mortalities)

9. (Mortality, Differential) OR (Differential Mortalities) OR (Differential Mortality) OR (Mortalities, Differential) OR (Mortality Determinant)

10. (Mortalities)

11.4 or5 or 6 or 7 or 8 or 9 or 10

12. MeSH descriptor: [Infant] explode all trees

13. (Infants)

14.12 or 13

15.3 and 11 and 14 185
